# Abnormal respiratory progenitors in fibrotic lung injury

**DOI:** 10.1186/s13287-022-02737-y

**Published:** 2022-02-07

**Authors:** Ting Xie, Heather Lynn, William C. Parks, Barry Stripp, Peter Chen, Dianhua Jiang, Paul W. Noble

**Affiliations:** Division of Pulmonary and Critical Care Medicine, Department of Medicine, Women’s Guild Lung Institute, Cedars-Sinai Medical Center, Los Angeles, CA USA

**Keywords:** Lung, Fibrotic injury, ATII transition cells, Persistent epithelial progenitors

## Abstract

Recent advances in single-cell RNA sequencing (scRNA-seq) and epithelium lineage labeling have yielded identification of multiple abnormal epithelial progenitor populations during alveolar type 2 (ATII) cell differentiation into alveolar type 1 (ATI) cells during regenerative lung post-fibrotic injury. These abnormal cells include basaloid/basal-like cells, ATII transition cells, and persistent epithelial progenitors (PEPs). These cells occurred and accumulated during the regeneration of distal airway and alveoli in response to both chronic and acute pulmonary injury. Among the alveolar epithelial progenitors, PEPs express a distinct *Krt8*^+^ phenotype that is rarely found in intact alveoli. However, post-injury, the *Krt8*^+^ phenotype is seen in dysplastic epithelial cells. Fully understanding the characteristics and functions of these newly found, injury-induced abnormal behavioral epithelial progenitors and the signaling pathways regulating their phenotype could potentially point the way to unique therapeutic targets for fibrosing lung diseases. This review summarizes recent advances in understanding these epithelial progenitors as they relate to uncovering regenerative mechanisms.

## Introduction

**Fig. 1:**
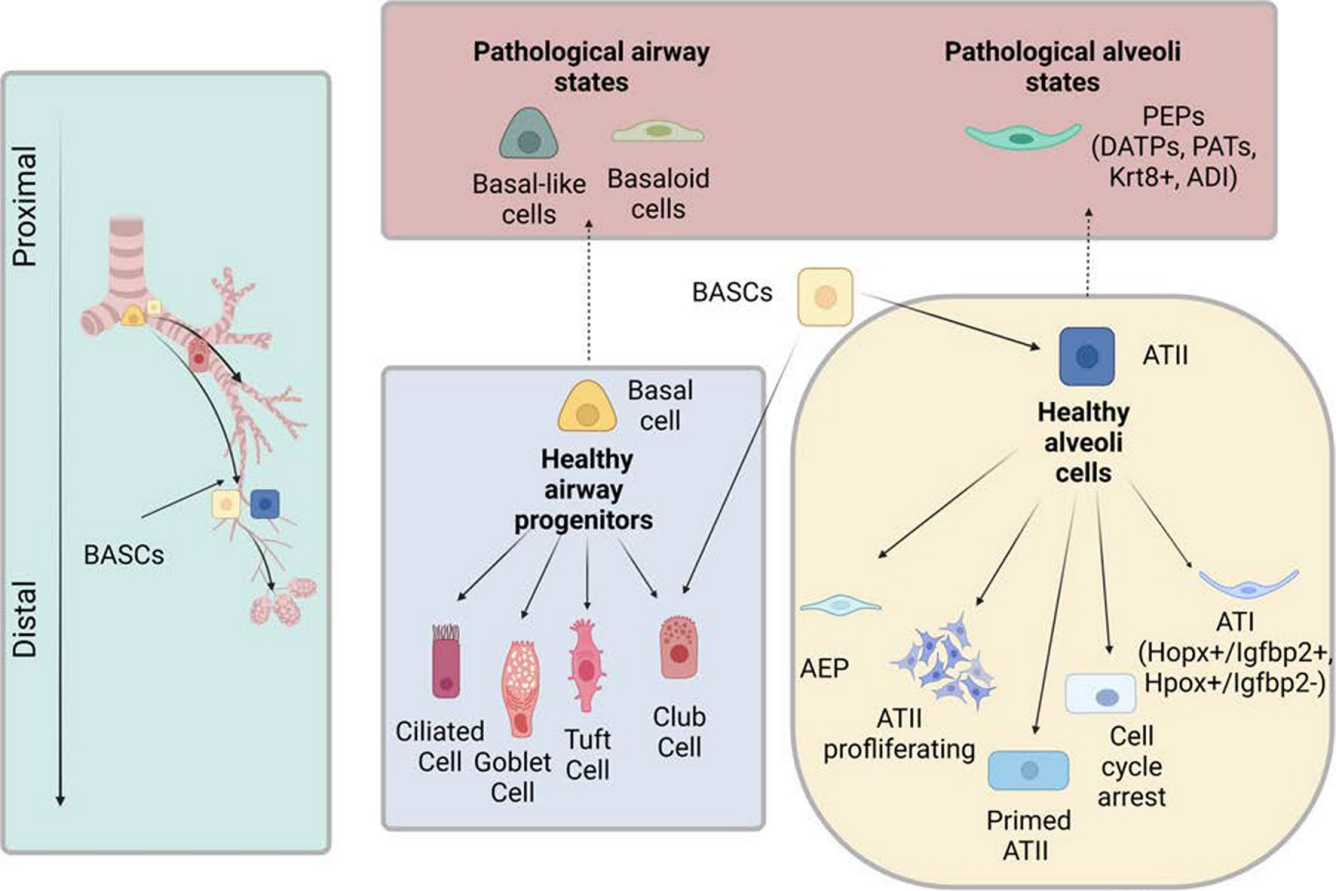
Airway progenitors migrated from the proximal airway to the distal airway. In the healthy airway, basal cells differentiate into ciliated, goblet, tuft, and club cells. In pathogenic states, basal-like and basaloid cells populate the airway. BASCs differentiate into club and ATII cells in the middle airway. ATII cells differentiate into AEP, ATII proliferating, ATII primed, cell cycle arrest, and ATI cells. In pathological alveolar states, ATIIs differentiate into PEPs (DATPs, PATs, Krt8 + , and ADI). This figure is created with BioRender.com

In normal lung, epithelial cells are the key components for both environmental barrier and gas exchange function [1]. Diverse populations of epithelial cells populate upper, mid, and lower airways. In the trachea and proximal conducting airways, the predominate epithelial cells are secretory, goblet, ciliated, and club cells (Fig. 1). In the terminal bronchiolar epithelium, neuroendocrine and basal cells (BCs) are present, along with BASCs. In the alveoli space, thin, flat ATI cells and cuboidal ATII cells are the two primary epithelial populations [2]. Depending on the location within the lung and the severity of injury, different progenitor populations respond to restore the damaged epithelium. Club cells are mainly found in the bronchioles, and they self-renew, give rise to new ciliated cells [3], and contribute to proximal airway repair. BCs have extensive proliferative potential, self-renewal capacity, and the capability to differentiate into club cells and ciliated cells [4]. In the lower airways and alveoli, epithelial cell turnover is slow compared to other epithelial linings (e.g., intestinal and epidermal). Bronchioalveolar stem cells (BASCs) are progenitor cells that originate in the bronchioalveolar duct junctions (BADJs) and migrate down into the alveolar space [5]. BASCs, which co-express *Scgb1a1* and *Sftpc*, contribute descendants to both bronchioles and alveoli [5] and, as demonstrated by lineage tracing, include club cells, ciliated cells, ATII and ATI cells [6, 7]. ATII cells are responsible for both self-renewal and differentiation into ATI cells [8].

In the injured lung, ATII cells serve the critical role of replacing damaged and abnormal alveolar epithelial cells [9, 10]. Recent studies have identified ATII transition types (or PEPs) that occur as ATII cells differentiate into ATI cells. During homeostasis and normal repair, PEPs do not accumulate or contribute significantly to the total number of alveolar epithelial cells, but rather efficiently complete their transfer into ATI cells. However, under certain disease conditions, such as idiopathic pulmonary fibrosis (IPF), PEPs accumulate and do not effective repopulate ATI cells [10–12], a defect that would contribute markedly to impaired respiration.

In this review, we will discuss these diverse epithelial progenitor lineages, the signaling pathways involved in controlling the fate of these progenitors, and the diseases and genetic predispositions in which alterations in their behavior contribute to maladaptive injury repair mechanisms. We also discuss current research approaches that are used to study these epithelial progenitors.

### Progenitors in alveolar niche and ATII transition cells/PEPs during alveolar regeneration

The alveolar space comprises over 70% of the lung and is the basic ventilatory unit involved in regulating gas exchange. In the adult lung, there are regional progenitor cell populations. BASCs are a specific type of progenitor that expresses both club cell marker SCGB1A1 and ATII cell marker SFTPC and are located at the BADJ [5]. BASCs contribute to both bronchiolar repair and alveolar repair and can give rise to club, ciliated, ATII, and, in turn, ATI cells. ATII cells are mainly account for alveolar repair (Fig. 1). Recent scRNA-seq and lineage tracing studies has suggested that BASCs can be segregated into two subpopulations (BASC-1 and BASC-2) [6], indicating that BACSs are a heterogeneous population. The BASC-1 subpopulation is enriched with *Cyp2t2* and *Scgb1a1* gene expression, while BASC-2 subpopulation is highly expressed with *H2-Ab1, CD74*, and *H2-Aa* genes. As *Scgb1a1* is a club cell maker and *CD74* labels ATII cells [13, 14], the sharing of gene expression signatures with club and ATII cells suggests the differentiation potential of the subpopulations of BASCs. BASC-1 might correspond to bronchiolar repair by differentiating into club cells, and BASC-2 may differentiate into ATII cells for alveolar repair, but these assumptions warrant further investigation. Using a *Sftpc*^+^ ATII depletion mouse model, it was concluded that there are more *Sftpc*^+^ ATII cells derived from alveolar ATII cells than from BASCs [15]. However, a recent study found that mouse *Scgb1a1* derived *Sftpc*^+^ BASCs (also termed secretory ATII cells, sAT2 cells) are capable of long-term self-renewal, more so than residual AT2 cells in in vitro organoids [16]. It remains unknown in lung fibrosis if ATII cell depletion occurs within fibrotic area or if BASCs or the remaining ATII cells are the major source for regeneration of ATII cells in the alveoli.

Besides BASCs, studies have shown that club cells [3], BCs, integrin α6β4 positive cells, H2-K1 high, and Uroplakin3a (*Upk3a*) expressing airway epithelial progenitors can migrate to the alveoli and give rise to ATII cells under conditions of severe injury that involve broad epithelial denudation [17–21]. An abnormal cluster of *Fstl1*^+^ secretory cells were found during defective club cells (or secretory cells) transition to ATII cells [16]. These cells were marked by *Id3, Porcn*, and *Cdkn1c* and blocked the fate conversion from secretory to ATII cells when lacking sustained Notch activity. The assumption that these above-mentioned airway progenitor cells regenerate ATII cells is partially based on the structurally contiguous of airway and alveoli. It is assumed that the severity of the injury determines the degree that ATII cells derived from airway progenitors can be replenished. Consistently, in other organs, ductal epithelial cells can contribute to functional unit progenitor cell regeneration, such as β cell in pancreas [22] and hepatocytes in liver [23].

A majority of the ATII cells utilize self-renewal to maintain the alveoli population during homeostasis of slow cellular cycling and in injury conditions of ATII cell loss [15, 24]. Alternatively, a population of facultative ATII progenitor cells (AEPs), which is Wnt-responsive and labeled by Transmembrane 4 L Six Family Member 1 (TM4SF1), were suggested to participate in alveolar regeneration [25]. AEPs also show distinct genetic profiles from ATIIs in lung development, including the import gene *Nkx2.1*. In addition, AEPs were shown to have ~ 40% of their chromatin open (via scATAC seq) compared to ATII cells.

Besides their self-renewal function, ATII cells can differentiate into ATI cells. Originally, it was thought that ATII cells are the only genitor of ATI cells. Except for the minor contribution of alveolar epithelial from bipotential progenitors [26, 27], there is no evidence that other airway progenitor cells can differentiate into ATI cells without initially differentiating into ATII cells. However, there is emerging evidence that ATII transition cells or PEPs occur during the differentiation of ATII cells to ATI cells, which were regarded as potential key characteristics of fibrotic injury. The ATII transition cells, previously described in nitrogen dioxide-induced lung injury [28], was recently found in pseudomonas aeruginosa injured and post-pneumonectomy ATII lineage labeled mouse lungs [29, 30]. These transition epithelial progenitors accumulated following injury in persistent Notch activation condition, expressed low levels of ATII and ATI cell markers, and were associated with retarded differentiation of ATII cells into ATI cells [29].

Furthermore, a subset of distinct ATII transition cells or PEPs population, damage-associated transient progenitors (DATPs), were characterized by gene expression of *Krt8*^+^, *Cldn4*, and *Lgals3*, and were extremely rare at steady state but arose during alveolar regeneration after single-dose bleomycin injury. DATPs accumulated following inflammatory signal IL-1β stimulation, blocked ATI differentiation, and impaired alveolar regeneration [10]. The DATPs subpopulation was also found in COVID19 patient lungs and were associated with a progressive decrease in ATI cell abundance [31]. In addition, a pre-alveolar type-1 transitional cell state (PATS) were observed during the differentiation of ATII cells into ATI cells. These PATS occurred in mouse lungs post-single-dose bleomycin injury and presented in the fibrotic regions of the idiopathic pulmonary fibrosis (IPF) lungs [12]. By using *Sftpc* ATII and *Krt19* lineage labeled mice, PATS that displayed a stretched morphology were shown to derive from ATII and *Krt19* lineage cells post-bleomycin injury [30].

**Fig. 2:**
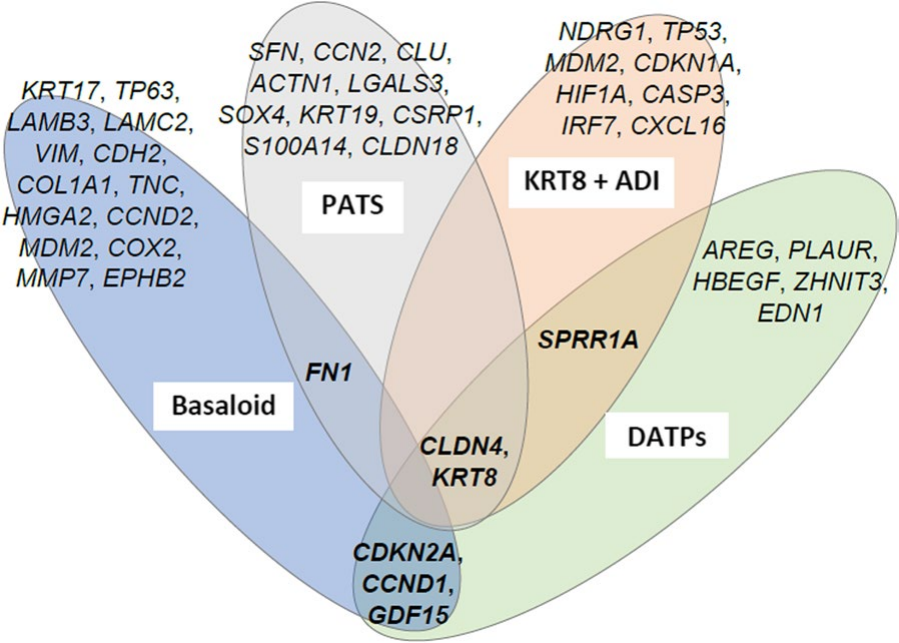
Venn diagram shows the types of published PEPs and signature marker genes. Genes labeled in bold represent overlapping markers. All genes are presented with the standard nomenclature for *homo sapiens* even if work was one in model organisms

Lastly, a unique *Krt8*^+^ alveolar differentiation intermediate (ADI) population was found during alveolar regeneration post-single-dose bleomycin lung injury, was derived from ATII cells, and might give rise to ATI cells as demonstrated by scRNA velocity analyses [11]. These abundant *Krt8*^+^ ADI belong to ATII transition cells or PEPs population and had stretched morphology (partially spread, but not cuboidal), features p53 and NFkB activation, and displayed transcriptional features of epithelial–mesenchymal transition (EMT) and cellular senescence. The *Krt8*^+^ ADI cells were also found to be persistently present in human fibrotic lungs and appeared in acute respiratory distress syndrome (ARDS) caused by influenza-A and pneumococcal infection as well as interstitial lung disease patients [11]. These reports showed, regardless of the different naming of PEPs that they occurred in different injury/disease settings with the similarities resulting from abnormal differentiation of ATII cells. It remains unclear whether, and under what conditions, the various PEPs might have the potential of completing terminal differentiation into ATI cells. Figure 2 summarizes the markers found in the previously discusses PEPs.

There are potentially two types of ATI cells in adult mouse lungs, as have been shown in post-pneumonectomy (PNX) alveolar regeneration [9]. Among this population of ATI1 cells, *Hopx*^+^/*Igfbp2*^+^ ATI cells are considered as terminal ATI cells while *Hopx*^+^/*Igfbp2*^−^ ATI cells are not. It is not fully understood whether these PEPs have the capacity to fully differentiate into terminal and functional ATI cells and what are the underlining mechanisms that drive the process.

There are other subclusters of ATII cells that have been shown by recent scRNA-seq analysis. For example, activated ATII cells marked by injury-induced genes (*Lcn2* and *Il33*) and Mki67 + proliferation clusters were found concurrently with *Krt8*^+^ ADI cells in bleomycin injured mouse lung [11]. In another study, ATII cells were segregated into proliferating, cell cycle arrest (senescence) and transdifferentiating (intermediate) ATII subpopulations in mouse lung 7 days post-LPS injury [32]. Primed ATII cells and cycling ATII cells were found concurrently with DATPs [10]. Further, stepwise differentiation trajectories were suggested from primed ATII cells to DATPs and then to ATI cells by scRNA velocity analysis. These studies started to explore the subpopulation of ATII cells and the interrelationship of the ATII subpopulations. It is hoped that the subpopulation of ATII cells and the differentiation checkpoints could be dissected in a deeper degree by multiple methods, especially in the human lung.

ATII self-renewal has been shown to be influenced by the mesenchymal cells in the alveolar niche/microenvironment [33, 34]. *Pdgfra*^+^ cells can facilitate the self-renewal and differentiation of ATII cells in vitro [15]; it is reported that *Axin2*^+^/*Pdgfra*^+^ cells supported ATII cell regeneration through functional signaling pathways, including interleukin-6 (IL6)/Stat3, Bmp, and Fgf pathways [15, 35, 36]. In addition, *Lgr5*-expressing mesenchymal cells promoted ATII cell differentiation [35]. Furthermore, fibroblasts provide short-range Wnt signals to neighboring ATII stem cells and maintain their stemness [24]. Hedgehog activation in lung mesenchymal fibroblasts led to reduced ATII regeneration and can be rescued by HGF in vitro [37]. Profibrotic mesenchymal cells retarded ATII cell growth and were associated with suppressed growth hormone receptor (GHR) expression in secreted vesicles [38]. These reports demonstrated that the impairment of ATII renewal in the chronically injured lungs could be due to the loss of supportive paracrine signaling from mesenchymal cells. The changes that occur in secreted supportive factors from mesenchymal cells during injury subsequently impact ATII renewal and make the disease more severe. It will be interesting to investigate whether niche supporting factors can influence the occurrence and differentiation of the PEPs.

### Involvement of airway progenitor cells for alveolar remodeling

BCs are speculated to be the major airway stem/progenitor cells and have been shown to differentiate into airway club cells, ciliated cells, tuft cells, and secretory goblet cells [39]. In cases of severe injury with massive depletion of ATII cells, BCs can migrate to lower airway or alveoli, become distal basal-like cells, and promote alveoli regeneration by differentiating into ATII and ATI cells [18, 21, 40]. BC expansion is gradually recognized as a common feature of epithelial remodeling, as originally discovered in influenza-induced mouse injury models as BC “pod” [40], and later in chronic lung diseases [41] and IPF [42] as BC hyperplasia, which might resemble a precancerous state [43]. BCs have traditionally been thought to line the airway basement membrane, to express markers including *KRT5*, *TP63*, and *NGFR*, and to be cuboidal in shape [44].

Recently, a type of basal-like cells predominantly accumulated at the edge of myofibroblast foci in IPF lungs, termed basaloid cells [45], might contribute to the airway epithelial-lined cysts known as “honeycombs” in the distal parenchyma. These “honeycombs” might contain this basaloid subtype of BCs that are secretory or goblet-like [46]. These basaloid cells are transcriptionally distinct from other epithelial cell types subclustered by scRNA-seq, and they express markers including *TP63*, *KRT17*, *LAMB3*, and *LAMC2*, but not *KRT5* and *KRT15*. They also feature EMT and senescence-related genes. These basaloid/basal-like cells might be able to seal the injured wound area by using their produced laminin [47], and these cells might produce serials of extracellular matrix and function as a sticking type of cell that pulls the mesenchymal cells and other epithelial progenitor cells together for sealing the injured wound [48].

Another assumption is that these basal-like/basaloid cells might be able to provide a basal membrane to help ATII progenitors reside prior to self-renew and/or further differentiate. A trial of transplantation of *Sox9*^+^ cells (including basal-like cells) into injured lung claimed to be able to improve the recipients’ (bronchiectasis patients) lung function [49]. These transplanted *Sox9*^+^ basal-like cells seemed to be incorporated into the bronchiolar region and gave rise to club cells and ATI cells in immunodeficient non-obese diabetic/severe combined immunodeficiency (NOD/SCID) mice post-bleomycin injury. *Sox9* was expressed in the tip of the distal epithelium and has been shown to regulate extracellular matrix (ECM) and cell movement. Loss of *Sox9* leads to defects in laminin deposition [50]. It is worth noting that these isolated *Sox9*^+^ basal-like cells have the capacity to proliferate massively in plastic culture and can be easily passaged. The massive proliferation feature might suggest they have acquired features of mesenchymal cells, which corresponds with the EMT feature noticed by scRNA-seq in IPF basaloid cells [45].

Senescence-related gene expression in the fresh sorted basaloid cells was found by scRNA-seq. It is assumed that at the stage in returning to pulmonary homeostasis, these senescent basaloid cells might be detected and be cleared by immune scavenger cells, such as macrophages [51]. Apparently in the IPF lungs, these senescent basaloid cells are persistent and pathogenically halt the regeneration of functional alveoli [52]. It is noticeable that the accumulation of basaloid cells were accompanied by elevated regulatory T cells [45], and regulatory T cells were found to contribute to tumor development and progression [53]. From this aspect, it is possible that accumulation of basaloid cells in the EMT in the early tumor microenvironment resembles a precancerous state.

A specific regional mesenchymal lineage *Gli1* + cells, located in airway epithelium niche, exhibit properties of mesenchymal stem cells (MSCs) [54]. *Gli1* + MSCs were shown to promote metaplastic differentiation of airway progenitors into *Krt5* + basal cells [55]. This study provides a functional link between the *Gli1* + MSC niche population and metaplastic basal cell transition of airway epithelial progenitors in fibrotic zones in mouse lungs [55]. It is not fully clear whether other mesenchymal cell populations play roles in the basal cell niche.

### Diverse airway and alveolar epithelial progenitors as new features of disease pathogenesis

These ATII transition cells/PEPs have been observed across chronic and acute fibrosis-related pulmonary diseases. The cellular characteristics and phenotypes of the abnormal epithelial progenitors varies by disease pathology. The PEPs we described above were mainly found in patients with IPF or in bleomycin-induced lung fibrosis mouse models and were considered as new features of IPF pathology. We will describe below the epithelial progenitors found in other fibrosis-related pulmonary diseases.

### Chronic obstructive pulmonary disease (COPD)

COPD is a progressive condition of chronic bronchitis, pulmonary obstruction, and emphysema. Fibrosis of small airways and respiratory vasculature has been established as a feature of COPD. There were aberrant basaloid cells found in COPD lungs, but these were far less prevalent than in the IPF lungs [52]. In another report, basal-like extra vivo clones, expressing markers *p63* and *KRT5*, were derived from distal epithelial progenitors obtained from lung tissues of COPD patients [56]. The pathological implications from these basaloid cells and basal-like clones suggests a possible link to the etiology and progression of the disease.

### Coronavirus disease 2019 (COVID19) infection

The leading cause of death from the COVID-19 virus is respiratory failure coupled with ARDS, and post-covid pulmonary fibrosis has been reported [57–59]. Studies of the recent COVID-19 lung cell populations found that hospitalized COVID-19 positive patients showed a significant decrease in total epithelial cells and global impairment of epithelial cell regeneration in the proximal and distal lung [31]. A population of PEPs with the *KRT8*/*CLDN4*/*CDKN1A* phenotype previously identified as DATPs were more frequent in the lungs of COVID-19 patients versus controls [10, 31]. The elevated levels of *Il1β* secreted from macrophages/monocytes in COVID-19 patients was speculated as the reason why this inflammation activated PEP, DATPs, were identified in high numbers. Whether PEP occurrences could be a driving force of the unremitting post-covid pulmonary fibrosis warrants further investigation.

### Influenza infection

Lung fibrosis was developed in some cases post-H1N1 influenza viral infection [60]. Chronic lung disease patients with frequent exacerbations of influenza infection subsequently developed fibrotic interstitial lung disease [61]. Following H1N1 influenza viral infection, an expansion of distal airway stem cells (DASC) expressing BC markers *Trp63* and *Krt5* assembled into nascent alveoli at sites of interstitial lung inflammation. Selective ablation of the *Krt5*^+^ DASCs in vivo prevented this remodeling, leading to pre-fibrotic lesions and deficient oxygen exchange [21]. These basal-like DASCs have the potential to differentiate into ATI cells, ATII cells, and bronchiolar secretory cells, which is supported by the evidence that transplantation of DASCs diminished the structural changes in the infected lungs [21]. Similarly, another study suggested that *p63*^+^/*Krt5*^+^ basal-like lineage-negative epithelial progenitors (LNEPs) cells were activated after influenza or bleomycin injury in mice, and these *Scgb1a1*^−^/*integrin α6β4*^+^ LNEPs proliferated and migrated to injured alveoli, exhibiting differentiation potential towards mature ATII cells, and potentially contributing to the micro-honeycombing structure that characterizes progressive fibrotic lung disease [19, 40]. More recently, a population of ectopic solitary chemosensory cells (SCCs), expressing tuft cell marker DCLK1 and derived from *p63*^+^/*Krt5*^+^ BCs by lineage labeling, appeared in the areas of remodeled alveoli post-A/H1N1/PR/8 influenza viral infection [62]. These influenza activated basal-like cells, and activated BCs seems to have the ability of differentiation towards alveolar epithelial cells, but it is uncertain at which disease stage these cells will start to differentiate into the alveolar epithelial cells.

### Bronchopulmonary dysplasia (BPD)

BPD is a chronic lung disease that usually occurs in premature infants. The pathologic findings of BPD includes airway fibrosis and interstitial fibrosis [63]. Alteration of the composition of alveolar epithelial cells were found in hyperoxia injured mouse models that represented the BPD phenotype [64]. This recent scRNA-seq analysis for hyperoxia mouse lung revealed an ATII *lyz1*^+^ population as a subpopulation of ATII cells. *lyz1*, an encoding lysozyme with decreased secretion in BPD [65], was found to be a marker of the activated ATII subpopulation [11] and enriched in ATII ^late^ (a stage right before becoming a mature ATII cell) in another scRNA-seq study for embryonic mouse lung [27]. The similarity of marker for ATII transition cells or PEPs found in different diseases or different cell stages implicates the immature or defect condition of ATII cells in pathology of certain diseases.

### Recent findings on signaling pathways involved in epithelial progenitors

The occurrence and differentiation of diverse types of epithelial progenitors involve similarly prevalent signaling pathways under different injury conditions. Here we highlight the WNT/β-catenin, TGFβ, Notch, cellular senescence, and inflammatory-related signaling pathways.

### WNT/β-catenin signaling

WNT/β-catenin signaling pathway is essential for lung development and homeostasis of progenitor ATII function [24, 25], and activation of WNT/β-catenin signaling facilitates the repair and regeneration of the lung after injury [34, 66]. Recently, it was reported that WNT/β-catenin signaling can be induced by blockage of LTβR signaling in ATII cells, which leads to enhanced lung alveolar regeneration in chronic smoking-induced lung diseases such as chronic obstructive pulmonary disease (COPD) [67]. Furthermore, WNT/β-catenin signaling induced by hypoxia was reported to promote the ATII reconstruction and blockade the *Krt5*^+^ basal-like activation from LNEPs for alveolar regeneration in influenza injured mouse lungs [19]. In another influenza study, facultative Wnt-responsive AEPs were shown to contribute to functional alveolar epithelial regeneration by generating both ATII and ATI cells after injury [25]. Furthermore, in an in vitro study, Wnt activation was shown to increase ATII self-renewal, while Wnt inhibition enhanced ATI differentiation in both human and mouse ATII organoids [25]. These observations indicated the beneficial roles of WNT/β-catenin signaling for ATII cell mediated alveolar regeneration in different conditions post-injury. In regard to PEPs, chronic activation of WNT/β-catenin signaling (7 day WNT3A treatment in vitro), but not WNT5A or acute WNT3A (24 h treatment) stimulation, has been found to induce cellular senescence and *Krt8* expression (PEP features) in primary murine ATII cells in vitro [68]. These data suggest chronic WNT3A signaling might enhance the production of PEPs from ATII cells in vitro by activation of the canonical WNT/β-catenin pathway. It is assumed that WNT/β-catenin signaling spatiotemporally regulates the ATII self-renewal and affects ATI differentiation, while chronic activation of canonical WNT/β-catenin signaling might promote ATII-to-PEP transition.

### TGFβ signaling pathway

TGFβ1 has been long recognized as a growth factor that is elevated in pro-fibrotic conditions. TGFβ1 was enriched in aberrant basaloid cells lined by myofibroblast foci in IPF lung tissue, which might be activated locally through their integrin repertoire [52, 69]. A *TGFB1*^hi^ *CTHRC1*^+^ mesenchymal population were found adjacent to *KRT17*^+^/*KRT5*^−^ basaloid cells (also termed ABIs) [70]. Suppression of TGFβ induced BCs reentry into the cell cycle and initiates epithelial regeneration via enhancing *Id2* expression [71]. Activation of TGFβ signaling was found in IPF HTII-280^+^ ATII cells by scRNA-seq analysis [72], and TGFβ signaling might be responsible for halting ATII self-renewal and inducing ATII differentiation into ATI cells in LPS-induced acute lung injury. TGFβ signaling was highly upregulated in the cell cycle arrested ATII subpopulation and relatively downregulated in ATII transdifferentiating cells detected by scRNA-seq analysis [32]; this suggests TGFβ signaling is required for ATII cell cycle arrest and ATII differentiation into ATI cells.

Another report suggested that TGFβ signaling is highly activated during early differentiation of ATII into ATI cells, and subsequent TGFβ deactivation promotes late differentiation. They also found that *Krt8* and *Krt18* were upregulated during ATII differentiation into ATI cells in vitro, and inhibition of TGFβ signaling attenuates *Krt8*/*Krt18* upregulation [73], suggesting that TGFβ signaling might contribute to ATII transition cells/PEPs formation, thus recapitulating a failure of TGFβ signaling deactivation that may result in the persistence of ATII transition cells and PEPs in IPF [12, 73]. Indeed, another study found that TGFβ signaling was upregulated in a presumably ATII transition cell subpopulation accumulated in PNX-treated *Cdc42*-null mouse lungs and was responsible for mechanical tension driven periphery-to-center progression of lung fibrosis in a spatially regulated manner [30]. These studies elaborated on the pivotal role of TGFβ signaling in basaloid cells, BCs, and in ATII transition cells/PEPs during ATII differentiation into ATI cells. Specifically, early activation of TGFβ enhances PEP marker expression in ATII cells, and subsequent suppression of TGFβ promotes differentiation into ATI cells. However, it seems obvious to speculate but still no experimental verification has determined whether it is the myofibroblast secreted TGFβ that activates the integrin receptors on ATII cells that stimulates ATII-to-PEP transition with subsequent PEP accumulation.

### Notch signaling pathway

Airway homeostasis can be easily disrupted by endogenous and exogenous insults, such as chronic micro-wound scarring, acute hypoxemic injury, pseudomonas-induced acute lung injury, and H1N1 infection in the lung. Elevated Notch signaling was found in collapsed alveoli post-hypoxemia and H1N1 infection, as well as in fibrotic lungs [19, 29, 42]. It is reported that Notch signaling initiates the expansion of LNEPs to basal-like *Krt5*^+^ cells in an alveolar remodeling following H1N1(PR8) influenza infection in a *HIF1A* dependent manner. Further, Wnt activity antagonized Notch signaling to favor LNEPs differentiation into ATII cells for recovery [19, 29]. In addition, Notch inhibition skews the differentiation of human secretory cells (*CC10*^+^*KDR*^+^*HTII280*^−^) into ATII cells instead of ciliated cells and BCs in culture conditions [16]. Recent scRNA-seq mapping of epithelial cell types for normal and IPF human airways suggested that BC subpopulations constitute a hierarchy regulated by Notch signaling. Within this hierarchy, Notch receptor, Notch2, maintains undifferentiated in BCs and restricts basal-to-ciliated differentiation, and Notch3 restrains BC secretory differentiation [42]. Furthermore, deletion of *Dlk1* (a non-canonical Notch ligand) in ATII cells induced persistent Notch activation and led to accumulation of ATII transition cells following pseudomonas injury. These findings suggested the cell fate selection feature of Notch signaling in the subpopulations of basal and ATII cells. Suppression of Notch facilitates ATII cell recovery, while persistent Notch activation promotes ATII transition cells or PEPs. It will be interesting to investigate whether this phenomenon is also representative of PEPs in the fibrotic lung.

### Cellular senescence pathways

Another common signaling pathway found in PEPs is cellular senescence. PEPs in fibrotic lung, including Basaloid cells, DATPs, PATs, and *Krt8*^+^ ADI cells, all display highly expressed genes related to senescence [10–12, 52]; this suggests that senescence not only is a feature for fibrotic fibroblast [74, 75], but represent a PEP stage halting full ATII differentiation into ATI cells in fibrotic lungs. These PEP senescence-related genes include *CDKN1A*, *CDKN2A*, *CCND1*, *CCND2*, *MDM2*, and *GDF15* [52]. In the same line, loss of *Sin3a* in ATII cells has been shown to initiate a program of p53-dependent cellular senescence, ATII cell depletion, and spontaneous, progressive pulmonary fibrosis [76], but whether *Sin3a*-null ATII cells represent PEPs is currently unclear. In another study, senescent marker *CDKN2A* was overlapping with the basal epithelial markers *Krt5* and *Krt17*, which localize to bronchiolized epithelial structures in fibrotic regions of IPF and systemic sclerosis-associated interstitial lung disease (SSc-ILD) lung tissue [77]. Whether senescence can be a reversible transient state accompanying tissue regeneration in fibrotic lung is currently unclear. It is challenging and the tools to lineage label senescent cells [78] are being developed to presumably able to resolve this issue.

### Inflammatory-related signaling pathways

Recent studies have provided evidence that indicate inflammatory signals trigger ATII cells response to injury. IL6 has been shown to promote ATII cell self-renewal in an innate immune receptor TLR4 dependent manner at an early stage of fibrotic lung injury [79]. It is reported that macrophage derived IL1β activated a subpopulation of primed ATII cells expressing IL1R1 for differentiation into DATPs, while chronic IL1β halted DATPs transition into functional ATI cells, which results in impaired alveolar regeneration in fibrotic lung [10]. The same group did follow-up research and found that IL1β signaling through IL1R1 on ciliated cells via paracrine Notch ligands, *JAG1* and *JAG2,* is essential for secretory cell differentiation of ATII cells [16]. These studies suggest that inflammatory signals are required for ATII cell mediated alveolar regeneration but sustained inflammatory response can cause defects for generating functional alveolar niche following injury. These inflammatory signals can function directly on the effected ATII cells, or indirectly on upstream epithelial cells, thus executing a sequential effect. A recent study that transplanted human ATII cells into bleomycin-induced fibrotic lungs of NOD scid gamma immunodeficient (NSG) mice revealed ATII-to-basaloid/basal cell transition [70]. It is intriguing to see whether, in fibrotic human lung where inflammatory signals present, these inflammatory signals are required for ATII-to-basal cell transition.

### Up-to-date approaches for researching epithelial progenitors

#### Single-cell RNA (scRNA)

ScRNA-seq from multiple companies and across several technology platforms has been utilized to understand epithelial progenitors. The expansion of single-cell sequencing into MULTI-seq and spatial RNA-seq has been further used to determine the epithelial progenitor subpopulations and their gene expression characteristics [27, 45, 64, 80]. A stem cell progenitor hierarchy in the distal airway has been well documented in the previous literature [1, 15, 33, 72]. Additionally, scRNA-seq has allowed for greater probing of heterogeneous airway and alveolar epithelial progenitor types and with the combination of cell lineage label produced convincing evidence for determining several types of PEPs [10, 12, 52].

### Lineage tracing mouse tools

Lineage-traced mouse tools for lung epithelial cells are well described elsewhere [81]. Here we updated the recent used epithelial cell type-specific mouse lines that have been used in studying the PEPs. There are many diverse mouse lineage tracing tools, and we have included short summaries of the most recent, novel, and pertinent to airway and alveolar epithelial cells (Table 1), and we have specifically organized these by PEP lineages, including DATP, PATS, and BC lineages (Table 2).

**Table 1:** Lineage-traced mouse tools for epithelial specific mouse lines

Strain	Lineage	Markers
*Shh*^*Cre*^ [19, 71, 82]	endodermal epithelial marker	*Scgb1a1*^+^ club epithelial cells in the proximal airway, with scattered expression in ciliated epithelium and Sftpc^+^ alveolar type II epithelial cells [82]
*Sox9*^*CreER*^ [83] and Sox9^cre/+^ [84]	distal airway epithelial cells	*Sox9* lineage cells can give rise to ATI and ATII cells after E13.5
*Sox2*^*CreER*^ [18, 19]	proximal airway lung epithelial cells	the majority of *Sox2* lineage alveolar cells exhibited ATII marker *Sftpc* expression; *Sox2* lineage labeled airway epithelial cells are basal, secretory, and ciliated epithelial cells in the airway of adult lung, but not alveolar cells [44]
*Nkx2.1*^CreER^ [71]	both distal alveolar and proximal airway epithelial cells	*Nkx2.1* lineage labeled cells were strictly epithelial cells, which co-stained with pan-epithelial marker EPCAM [85]
*Upk3a*^CreER^ [17]	lineage labeled cells are progenitors that give rise to club cells and ciliated cells in the postnatal period	Adult *Upk3a* lineage labeled cells were distributed in the airway, clustered around neuroepithelial bodies (NEBs) and located close to BADJs
*Igfbp2*^CreER^ [9]	ATI cells after postnatal day 1 (p1)	In this study, 95% of the *Hopx*^+^ ATI cells express *Igfbp2* at p60. *Hopx*^+^*Igfbp2*^+^ cells are proposed to represent terminal differentiated populations of ATI cells
*Sftpc*^DreER^ [6, 7]	lineage label BASCs	Tamoxifen exposure will enable recombination of Dre and Cre, which results in labeling of *Sftpc*^+^/*Scgb1a1*^+^ BASCs. Dre and Cre mouse lines were used by cross with R26-RSR-LSL-tdTomato [6], R26-comfetti2 [86], and R26-TLR [7] reporter mouse lines

**Table 2:** Lineage-traced mouse lines specific to PEP lineages

Strain	Lineage	Markers
*DATP specific lineages*		
*Ndrg1*^CreER^ [10]	expressed in DATPs during alveolar regeneration	*Ndrg1* lineage labeled cells emerged with a majority of *Krt8*^+^ cells in the alveolar region post-bleomycin injury (day 9). There were 30% of ATI cells derived from *Ndrg1* lineage labeled cells post-bleomycin injury (day 28)
*Krt8*^CreER^ [10]	*Cldn4* + DATPs post-bleomycin injury	*Krt8* expression was only detected in *Cldn4*^+^ DATPs post-bleomycin injury (day 9) in the alveolar region and was prominent in *Pdpn*^+^ ATI cells post-bleomycin injury (day 28)
*Il1r1*^CreER^ [10]	expressed in airway ciliated cells and small subsets of mesenchyme cells in uninjured lungs	About 15% ATII cells were lineage labeled by *Il1r1* in uninjured lung, *Il1r1* lineage labeled ATII cell increased to 60% post-injury (day 14); 80% of the DATPs were lineage labeled by *Il1r1*
*PATS specific lineages*		
*Krt19*^CreER^ [12]	a transition population between ATII and ATI	*Krt19* lineage labeled cells co-expressing ATI marker AGER were found 12 days post-bleomycin. No *Krt19* lineage cells were found to give rise to *Sftpc* expressing ATII cells
*Ctgf*-GFP [12]	found in fibroblasts, but not in alveolar epithelial cells in normal lung	GFP expression in epithelial cells co-labeled by PATS markers *Cldn4*, *Lgals3*, and *Sfn* post-bleomycin injury and co-expressed low levels of ATII marker *Sftpc*
*BCs*		
*Krt17*^CreER^ [71]	transition population in the BC lineage	*Krt17* lineage labeled cells showed limited expression pattern in BCs in developing trachea. About 90% of the *Krt17* lineage labeled cells committed toward BC lineage at E14.5 and started expressing a mature BC gene signature *Krt5* at E16.5
*p63*^CreER^ [19]	Basal-like cells	It is reported that all arising *Krt5*^+^ cells post-influenza infection were lineage labeled by *p63*
*Krt6*-DTR [21]	Basal cells	This line enables the ablation of *Krt6* expressing cells upon diphtheria toxin treatment. A study showed that 90% loss of *Krt5*^+^ cells and over 99% loss of *Krt6*^+^ cells were found in *Krt6*-DTR mouse lung compared to wild-type control 15 days post-influenza infection
*Scgb3a2*^DreER^ [80]	Basal cells	This line enable lineage labeling of distal airway secretory cells, interlobar serous (IS) cells. This study showed IS cells became BCs following influenza infection

### Epithelial progenitor transplantation for treating clinical fibrotic lung diseases

It is thought that replacing the pathologic epithelial cells by exogenous progenitors is a promising treatment for fibrotic pulmonary diseases. Investigators have transplanted ATII cells, fresh isolated from healthy rat or differentiated from induced pluripotent stem cells (iPSCs) for bleomycin-induced mouse lung fibrosis, and found that transplanted ATII cells can be engrafted, reduce collagen deposition in the lung, and abrogate pulmonary fibrosis [87–89]. Adult rat lung spheroid cells containing mixed epithelial progenitors were reported to attenuate rat lung fibrotic progression [90]. Furthermore, ATII cells isolated from deceased organ donors were transplanted intratracheally in progressive IPF patients and were well tolerated [91]. More recently, human *SOX9*^+^ BCs expanded from bronchoscopic brushing were reported to give rise to alveolar and bronchiolar epithelium post-transplantation into bleomycin injured mouse lung and were found to enhance the recipients’ lung functions. Transplantation of autologous *SOX9*^+^ BCs enhanced lung tissue repair and lung function in two patients with bronchiectasis [49]. More mechanistic research is needed to validate the potential of epithelial progenitor transplantation in vivo in patients with lung fibrosis and other lung diseases. Long-term follow-up should be conducted to evaluate the tumorigenic potential of the exogenous or endogenous lung epithelial progenitors.

## Conclusions, current challenges, and prospects

The existence of epithelial progenitors in the distal airway with regenerative properties (both migration into and proliferation in the alveolar space) is established [5, 92]. Wnt/β-catenin, Notch, TGFβ, and senescence pathways have been shown to regulate ATII transition cells/PEPs under diverse causes and driver of lower airway injury [19, 32, 40, 68, 72, 76]. ScRNA-seq in combination with lineage tracing has helped to show that there are distinct ATII transition cells/PEPs, and while *Krt* expression is crucial, what combination of Krt genes (*Krt8*, *Krt14*, and *Krt17* have all be shown to be present in vitro transition states and utilized in vivo models) defines these ATII transition cells/PEPs and at what cellular checkpoints is unsettled. The dominant immunological, senescent, or hypoxic signaling pathways present in the alveolar microenvironment may determine what specific ATII transition cells/PEPs drives regeneration and repair in the alveoli. Further lineage-based studies to determine how these transition cells/PEPs originate may clarify these states.

Efforts to integrate the many single-cell seq datasets into a functional database has been an ongoing project in IPF research. Differentiating between PEP phenotypes and defining transition states makes utilizing multiple datasets from different research groups a challenge. An initiative to standardize single-cell terminology or single-cell taxonomy could aid in the creation of this database. Further experimentation may provide a more accurate framework for how to categorize transition state cell populations in IPF.

## Data Availability

Not applicable.

## Abbreviations

ScRNA-seq: Single-cell RNA sequencing
ATII: Alveolar type 2 cell
ATI: Alveolar type 1 cell
PEPs: Persistent epithelial progenitors
BASCs: Bronchioalveolar stem cells
BADJs: Bronchioalveolar duct junctions
Upk3a: Uroplakin3a
TM4SF1: Transmembrane 4 L six family member 1
DATPs: Damage-associated transient progenitors
PATS: Pre-alveolar type-1 transitional cell state
IPF: Idiopathic pulmonary fibrosis
ADI: Alveolar differentiation intermediate
ARDS: Acute respiratory distress syndrome
PNX: Pneumonectomy
IL6: Interleukin-6
GHR: Growth hormone receptor
NOD/SCID: Non-obese diabetic/severe combined immunodeficiency
ECM: Extracellular matrix
EMT: Epithelial–mesenchymal transition
COPD: Chronic obstructive pulmonary disease
LNEPs: Lineage-negative epithelial progenitors
AEPs: ATII epithelial progenitors
SSc-ILD: Systemic sclerosis-associated interstitial lung disease
COVID19: Coronavirus disease 2019
DASC: Distal airway stem cells
SCCs: Solitary chemosensory cells
BPD: Bronchopulmonary dysplasia
NEBs: Neuroepithelial bodies
IS: Interlobar serous
iPSCs: Induced pluripotent stem cells
BCs: Basal cells
